# Teachers’ Innovative Work Behavior as a Function of Self-Efficacy, Burnout, and Irrational Beliefs: A Structural Equation Model

**DOI:** 10.3390/ejihpe13020030

**Published:** 2023-02-07

**Authors:** Angelos Gkontelos, Julie Vaiopoulou, Dimitrios Stamovlasis

**Affiliations:** 1 School of Philosophy and Education, Aristotle University of Thessaloniki, 54124 Thessaloniki, Greece; 2 Department of Education, University of Nicosia, Nicosia 2417, Cyprus; 3 School of Psychology, Aristotle University of Thessaloniki, 54124 Thessaloniki, Greece

**Keywords:** innovative work behavior, self-efficacy, burnout, irrational beliefs, structural equation modeling

## Abstract

Teachers’ innovative work behavior (TIWB) is crucial in the contemporary demanding educational environments for overcoming any commonplace issues and to ensure sustainability and development. It refers to a process in which the employee tries to create new ideas, adopt them, apply them in the school context, and then communicate them to other members of the organization in order to achieve a communal benefit. Among a plethora of factors that could influence such behavior, self-efficacy, burnout, and irrational beliefs have been proposed as potential covariates. In the present study, the associations of the above constructs with TIWB are concomitantly investigated by employing structural equation modeling (SEM). Data were taken from the participation of in-service teachers (*N* = 964) in primary education via self-reported questionnaires. The proposed SEM model exhibited a satisfactory goodness-of-fit to the empirical data, highlighting the direct effects of the independent variables on TIWB, while mediation analysis showed that irrational beliefs and burnout act also as mediators between self-efficacy and innovative work behavior. The findings are in line with previous reports and are interpretable in the context of modern theories, while a discussion on theoretical and practical implications along with emerging ideas and perspectives for further research is provided.

## 1. Introduction

In today’s society of knowledge and technology, the school has to play a significant two-fold role, the first aspect of which is to support students’ knowledge acquisition, while the second is to cultivate critical thinking. A central element to this process is the available human resources. The challenges arising from multicultural issues, parental concerns, and leadership demands set a new agenda for teachers’ professional development, and innovation becomes a necessary condition for a sustainable school environment.

The transcendence of each commonplace and the adoption of novelty, however, requires the development of innovative work behavior (IWB), an iterative process in which the employee strives to find new ideas in order to adopt and promote them to other members of the organization, thus, achieving a shared benefit [1,2,3]. Although the development of innovative behavior within the workplace could be the springboard for the development of this organization, it is not a widespread feature of employees, while the cultivation of innovative ideas within the school organization does not imply the universal development of the innovative behavior of its members. In this process, teachers’ perceptions, motivations, and beliefs are crucial factors that could act as facilitators or inhibitors to the development of their IWB [4,5]. For example, individuals often possess views known as irrational beliefs, which usually act as a deterrent, bringing about non-functional consequences and, ultimately, influencing their actions and even their lifestyles. More specifically, irrational beliefs describe the structured notions of the individual about reality, are evoked by their daily experiences, and are the expressions of their behavior [6]. In addition, research has shown that the perceived self-efficacy of teachers affects their every activity in their professional career [7]. They refer to how competent the teachers feel about their ability to successfully complete their teaching work, as well as to manage their classroom by addressing the specifics of each student and encouraging their participation within the school context, while cultivating their characters and personality. Moreover, given increasing demands, the school environment is a place of enhanced work stress, which, in some extreme and recurring situational conditions, could lead to the teacher’s burnout, i.e., the situation in which the teacher is unable to adapt to an environment characterized by increased levels of work anxiety resulting in exhaustion [8,9].

Education, nowadays, encounters a great challenge for maintaining sustainable development in a quickly changing environment, which requires the procurement of aptitudes, skills, and information that are frequently missing from customary instructive programs. Moreover, there is continuous dispute and discussion about the quality affirmation of teaching, which reconsiders the change in instruction strategies, assessment methods, and their effect on teachers’ competencies [10]. The foremost advancements in instructional sciences emphasize the integration of the known reputable abilities, such as collaboration and communication with the novel talents for effective adaptability of educational programs and processes within the emergent demanding environments that set additional goals, such as a special objective of supporting students’ entrepreneurial capabilities [11]. Attaining innovative goals that guarantee a sustainable education system are undoubtedly facilitated by certain attentive teachers’ behaviors. A necessary condition for the implementation of innovations in the educational context is the positive teachers’ attitudes and their deeper understanding of how novelty is achieved and sustained [12]. This active educators’ role should be continuously supported to maintain a constant innovative work behavior, since studies have shown that even after a considerable period of time, teachers abandon the newly acquired behavior and return to comfortable old routines [13]. To this end, the policy-makers and stakeholders should encourage and reinforce teachers’ innovative work behavior, while training programs and basic curricula in university programs should cultivate such orientation to ensure that adaptability is an enduring success [14,15].

Acknowledging the importance of IWB and aiming to an illuminating contribution to the field, in this paper, teachers’ innovative work behavior is examined as a function of their self-efficacy, burnout, and irrational beliefs via multivariate statistical modeling.

### 1.1. Innovative Work Behavior

Innovation is a process characterized as a repetitive, complex, and nonlinear activity [16,17]. Understanding innovation in an organization is enabled by studying the stages of involvement that its members could follow [18]. Specifically, these stages include the conception of the new idea, the discussion among the members of the organization, the implementation, and the effort to transfer and disseminate the new idea in a broader context that goes beyond the framework of the organization. The above, Kanter’s proposed conceptual framework, has spurred research interest in exploring IWB and guided relevant inquiries [19]. However, there are varying definitions for IWB in the literature, indicating that the theory is not fully developed, and it is constantly being updated.

The common elements that characterize all endeavors explicated that IWB involves the deliberate creation, introduction, and implementation of new ideas within an organization targeting benefits to both the individual and the whole organization [20]. The individual behavior of the members of an organization aiming to introduce and implement new and useful ideas is an essential contribution to the development of the organization’s innovation [21,22,23], and comprise an iterative process of multiple stages in which employees conceive new ideas after exploration and ponder their promotion and sustainable implementation [2].

The above definition unveils the multidimensional nature of IWB. The underlying components have been determined as the above-mentioned stages, which have to be studied in tandem to draw valid conclusions [24]. Some points to be mentioned are that these dimensions have been found to be highly correlated [20] and, on the other hand, the IWB process encompasses overlapping stages in which the individual is expected to participate at any time [19]. Nevertheless, in research and practice, conditions that facilitate exploration and/or cultivation of innovation are favored when the dimensions are studied separately as distinct stages [17,18].

Among the prevailing approaches on teachers’ innovative behavior, a recent work proposes a conceptual framework that includes five dimensions, namely opportunity exploration, idea generation, idea promotion, idea realization, and idea sustainability [2]. This is accompanied by the relevant instrument (IWBS) for measuring these dimensions, which are implemented in the present research, after the proper adaptation [25].

### 1.2. Self-Efficacy

Teachers’ self-efficacy (SE) is defined as the beliefs they hold about their ability to teach successfully. They constitute their personal perceptions of their knowledge, the teaching methods they adopt, and the achievement of their students’ learning and behavioral performance [26]. As such, SE contributes to the completion of their work goals and the potential success of their work [27].

Teachers’ SE is reflected in everyday classroom activities related to student engagement, classroom management, and teaching, and has a direct impact on student performance [28,29,30]. Psychologically, SE is involved in the self-regulation process and is associated with other belief systems and influential variables, such as emotions [31,32,33].

For example, negative emotions are increased with reduced self-confidence and in the presence of confounder factors, such as irrational beliefs or dysfunctional beliefs, and can drastically affect thoughts, mental states, and behaviors on a daily basis [30,34]. Consequently, negative emotions in the classroom hardly allow teachers to build strong relationships of trust with their students or to successfully manage their classroom and, thus, these emotions often cause a sense of reduced SE [35].

Self-efficacy beliefs associated with emotional states are strong prognostic factors for job satisfaction and burnout situations [36], also affecting motivation and dynamically influencing teachers’ work [37], while acting as a mediator between burnout and self-perceived instructional competence [38]. It is also noteworthy that teachers who demonstrate higher resilience to the arisen problems are most likely to be those who possess strong positive beliefs about their abilities [39].

### 1.3. Burnout

Burnout (BT) is a crucial factor in workplaces, and it is traditionally considered to occur mainly due to emotional exhaustion [40,41], while the underlying process includes three stages, namely emotional exhaustion, depersonalization, and reduced personal accomplishment. In the present study, BT is defined as the result of increased demands on work in the face of reduced resources, leading to cognitive and emotional exhaustion and to gradual dismissal of work [42]. Furthermore, BT is described as a situation in which the teacher has difficulties trying to adapt to an environment with increased levels of work stress, while a simultaneous reduced stamina leads to exhaustion and disengagement from work [8,9].

Job BT has a negative effect on workers’ occupational health [43] by reducing job satisfaction [44] and increasing absenteeism [45]. Regarding learning outcomes and attainment of educational goals, BT causes a reduced performance in teaching, with negative effects on both the classroom climate and student performance [45,46,47] and, thus, has disastrous consequences for the quality of education. Job BT is influenced by self-efficacy [48].

Job demands–resources theory [49] was fostered as an appropriate framework for the present study of BT. The social and emotional demands that govern the teaching profession, along with teacher–student relationships, parent–colleague conflicts, and classroom climate, act as stressors associated with BT [50,51]. Limited available resources, ineffective school leadership, problematic behavior of the students, increased workload, the lack and pressure of time, and low income compared to highly demanding didactic work, can function as factors leading to exhaustion and progressive disengagement [46,52].

### 1.4. Irrational Beliefs

Irrational beliefs (IB) are unrealistic thoughts, lacking rationality and/or empirical support [53,54]. Their cause has not been clearly determined, since they might stem from both social factors, i.e., the individual’s education and cultural environment [55], and temperament traits, leading to general maladaptation [56].

Furthermore, IB are involved in appraising the environment organized in schemes [6], i.e., structures of objects that are attributed various values and traits in different evaluative circumstances. These IB are shaped by simple everyday experiences, yet they have a rather complex structure because they represent an individual’s understanding of the reality. This means that they are powerful enough to influence behavior and, in fact, constitute a specific type of evaluating perceptions, sometimes related to non-functional emotions and conditions [57]. It is empirically documented that emotional states that affect individuals’ thought processes and could disturb their self-control [58] might be associated with IB, i.e., thoughts that distort reality, making them negatively anticipate the outcome of a certain situation [54]. This is because IB produce dysfunctional myths, a special set of ideas about something, that may automatically cause involuntary negative emotions, ranging from a mild discomfort to denial or even depression [59]. Consequently, those emotions, in turn, may affect the influence of dysfunctional myths, in a dynamical feedback loop process, causing nonlinear behaviors [60]. The idiosyncratic nature of these kinds of beliefs has been noted in the reported difficulties in dealing with functional and dysfunctional thoughts [56].

Within the educational context, teachers’ IB could be beliefs about students’ misbehavior or a school’s unchangeable situations that are supposedly beyond any treatment. These evaluative perceptions combined can create latent views and decisions with dysfunctional behavioral consequences.

### 1.5. Previous Research

The literature in the field provides a documented background on the relationships between IWB and a number of individual differences and supports a rationale for further research.

Teachers’ SE has been shown as a factor which positively affects and enhances IWB [4]. At the same time, SE is related to their emotions, generating attitudes and beliefs about workplaces [31,33,61], while it is associated with burnout and signals teacher exhaustion [62]. Utilizing the job demands–resources theory, which distinguishes the dimensions of BT to exhaustion and disengagement, it was found that the job satisfaction, the pressure for the minimum time to complete the learning process [63], as well as critical reflection on the problems that arise during teaching [64], are potential predictors of IWB development acting as job demands [3]. On the other hand, the school environment, representing resources, plays an important role, and can have a positive effect on the development of IWB, along with favorable working conditions for the teacher [65]. In this context, professional autonomy [66] acts as a springboard for the emergent IWB.

Teachers’ IB may predispose them negatively and bring them face to face with several stressful situations, such as reduced confidence in their abilities and effectiveness and/or shaken trust in colleagues and the school system as a whole. Research findings have indicated the strong effect that IB have on teachers’ emotions, making them dysfunctional and reducing their work performance [67,68]. However, teachers show positive dispositions on interventions aimed at reducing these beliefs and facilitating the emergence of healthy emotions and functional behaviors [33,69]. Thus, it is reasonable that IB are often associated with teachers’ SE within the school unit [61]; nevertheless, notionally, they are considered independent and are examined separately. The creation of IB may be due to dysfunctional emotions, such as resentment, which are generated when new experiences conflict with SE, and a type of IB or their reinforcement is often expressed through the generalizations adopted by teachers about issues in their workplace [70].

Conclusively, research in the field has established at least the bivariate correlations between the aforementioned variables and IWB. Thus, new research and theory-driven inquiry is an endeavor seeking to explore the multivariate structure of associations among all the variables under study. That is to investigate concomitantly the effects and the relationships of self-efficacy, burnout, and irrational beliefs on innovative work behavior. This is the objective of the present endeavor, which is targeted towards theory development by providing a more realistic representation of the network-like relationships.

## 2. Materials and Methods

### 2.1. Research Hypotheses/the Proposed Model

**Figure 1:**
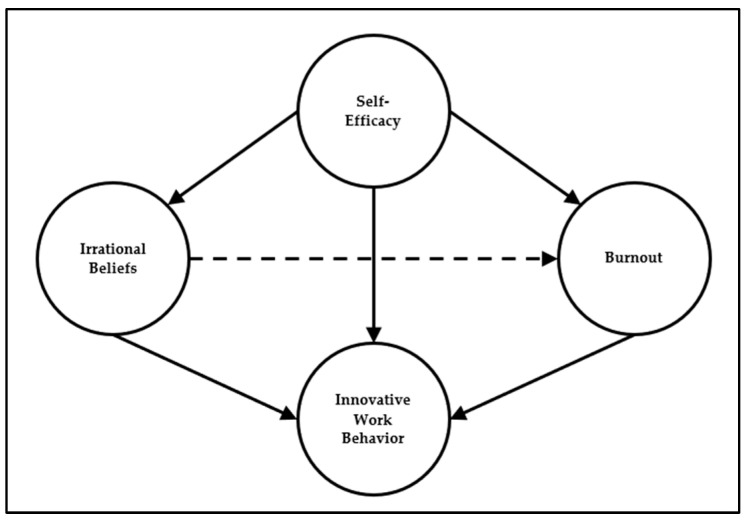
Schematic representation of the proposed model with burnout (BT) and irrational beliefs (IB) hypothesized as active mediators between self-efficacy (SE) and innovative work behavior (IWB), exhibiting direct and indirect effects.

Research on IWB has established certain relationships and effects, supported mainly via bivariate tests or linear regression analysis. However, bivariate correlations and restricted linear models ignore the potential effects of other variables being present. The complexity of the interactions among the determining factors demands a more realistic portrayal of their relationships. The positive effect of SE on IWB, along with the potentially negative effects of BT and IB, constitute a theory-driven set of hypotheses, which are sought to be explored concomitantly in a network of relationships. Figure 1 shows a schematic representation of the proposed SEM model, which embraces the research hypotheses regarding the relationships among the dependent and independent variables. The main feature of the model is that BT and IB are hypothesized as active mediators between SE and IWB, exhibiting direct and indirect effects. The research hypotheses are summarized as follows:

*H*_1_—SE has a positive effect on IWB.
*H*_2_—SE has negative effects on BT and IB.
*H*_3_—IB has positive effects on BT.
*H*_4_—IB has negative effects on IWB.
*H*_5_—BT has negative effects on IWB.
*H*_6_—IB and BT act as mediators between SE and IWB.

### 2.2. Participants

The sample of this study consisted of 964 primary education teachers, 77% of whom were female, with participant age varying from 22 to 65 years old (mean = 41.35, median = 40, SD = 10.88). The years of service of the participant teachers varied from 1 to 35 years (mean = 15.11, median = 14.5, SD = 10.63), while, in most cases, they served in schools located in a city (city = 60.27%, town = 16.28%, country = 23.44%). It is noteworthy that half of them (53.8%) held a master’s degree.

### 2.3. Procedure

The implemented self-completion questionnaire was uploaded on a web-based form via Lime Survey Forms, where the participants completed it anonymously. The procedure is characterized as opportunity sampling based on the teachers who were willing to participate and complete the questionnaire. The data collection was accomplished with the assistance of school principals from all primary schools in Greece, who disseminated and communicated an instructive e-mail to the in-service teachers, with a cover letter explaining the purpose and the confidentiality of the study, underlying its voluntary participation and its scientific objectives. The study has been approved by the institutional Research Ethics and Deontology Committee.

### 2.4. Instruments and Measures

Four questionnaires were implemented, corresponding to the four latent variables under investigation, which had all already been adapted to the Greek population, either by the other researchers or by the authors. Participants were invited to choose their degree of agreement on a 5-point Likert scale, were 1 indicates strong disagreement and 5 suggests strong agreement, respectively.

#### 2.4.1. Innovative Work Behavior Scale (IWBS)

The initial IWBS [2] proposes five dimensions, namely opportunity exploration, idea generation (sample item—asking critical questions about current situations at work), idea promotion (sample item—convincing others of the importance of a newly developed idea or solution), idea realization (sample item—testing solutions for unexpected problems that emerge, when putting ideas into practice), and idea sustainability (sample item—explicitly communicating the returns of the implemented idea outside the team), sorted into 44 items. The final adopted Greek version of IWBS did not include the “opportunity exploration” dimension, while 22 items from the initial model remained [25].

For the present measurements, confirmatory factor analysis (CFA) showed a satisfactory model-fit, as follows: [*χ^2^* = 396.85, *df* = 203, *p* < 0.001; *CFI* = 0.995; *TLI* = 0.994; *RMSEA* = 0.031; *90% CI of RMSEA* = [0.027; 0.036]; *SRMR* = 0.051; *NFI* = 0.990; *GFI* = 0.992].

Reliability measures of the four G-IWBS’s factors were computed using Cronbach’s Alpha (α) and McDonald’s Omega (ω), as follows: idea generation (IG) (*α =* 0.825/*ω* = 0.824), idea promotion (IP) (*α =* 0.891/*ω =* 0.891), idea realization (IR) (*α* = 0.911/*ω =* 0.914), and idea sustainability (IS) (*α =* 0.854/*ω* = 0.857). These reliability indices suggest that the present measurements with the G-IWBS sub-scales have a satisfactory degree of internal consistency.

#### 2.4.2. Teachers Self-Efficacy Scale (TSES)

For measuring SE, a teacher self-efficacy scale [26] was used. The instrument contains 24 items, examining 3 dimensions, namely efficacy in student engagement (sample item—How much can you do to get through to the most difficult students?), efficacy in instructional strategies (sample item—How much can you do to adjust your lessons to the proper level for individual students?), and efficacy in classroom management (sample item— To what extent can you make your expectation clear about student behavior?). The Greek version of TSES has been validated in previous research [71]. For the present measurements, reliability measures of the three TSES’s factors were computed using Cronbach’s Alpha (α) and McDonald’s Omega (ω), as follows: efficacy in student engagement (SES) (*α* = 0.782/*ω* = 0.783), efficacy in instructional strategies (SEI) (*α =* 0.757/*ω* = 0.757), and efficacy in classroom management (SEC) (*α =* 0.753/*ω =* 0.755), while CFA showed a satisfactory fit of the three-factor model [*χ^2^*(62) = 94.683, *p* < 0.005, *TLI* = 0.995, *CFI* = 0.996, *GFI* = 0.993, *NNFI* = 0.995, *RMSEA* = 0.023 (0.013–0.032), *SRMR* = 0.041].

#### 2.4.3. Oldenburg Burnout Inventory (OLBI)

For measuring teachers’ BT, the Oldenburg Burnout Inventory (OLBI) was used [72] which has been validated by its creators in the Greek population. The OLBI consists of 16 items classified in 2 dimensions, namely exhaustion (sample item—there are days when I feel tired before I arrive at work) and disengagement (sample item—it happens more and more often that I talk about my work in a negative way). For the present measurements, reliability measures of the two OLBI’s factors were computed using Cronbach’s Alpha (α) and McDonald’s Omega (ω), as follows: exhaustion (EXH) (*α =* 0.815/*ω* = 0.827) and disengagement (DIS) (*α* = 0.751/*ω* = 0.764), while CFA showed a satisfactory fit of the two-factor model [*χ^2^*(26) = 136.673, *p* < 0.001, *TLI* = 0.962, *CFI* = 0.972, *GFI* = 0.985, *NNFI* = 0.962, *RMSEA* = 0.056 (0.066–0.078), *SRMR* = 0.070].

#### 2.4.4. Teachers’ Irrational Belief Scale (TIBS)

The TIB scale was designed by Bernard [73] for evaluating teachers’ IB and includes four dimensions, namely self-downing (sample item—I really should be able to solve all of my students’ problems perfectly), authoritarianism (sample item—students should always be respectful, considerate, and behave well), demands for justice (sample item—one thing I find totally bad is the lack of communication between teachers and central administration), and low frustration tolerance (sample item—it is really bad to have to put in so many hours both inside and outside the class-room). The Greek version of TIBS contains 21 items, and it was validated and implemented in previous research [74]. For the present research, reliability measures of the four GTIB’s factors were computed using Cronbach’s Alpha (α) and McDonald’s Omega (ω): self-downing (SD) (*α* = 0.745/*ω* = 0.752), authoritarianism (ATH) (*α* = 0.760/*ω* = 0.772), demands for justice (DJ) (*α* = 0.684/*ω* = 0.684), and low frustration tolerance (LFT) (*α* = 0.735/*ω* = 0.741). Here, CFA showed a satisfactory fit of the four-factor model [*χ^2^*(183) = 587,155, *p* < 0.001, *TLI* = 0.953, *CFI* = 0.959, *GFI* = 0.974, *NNFI* = 0.953, *RMSEA* = 0.044 (0.042–0.052), *SRMR* = 0.058].

## 3. Results

Table 1 presents the descriptive statistics, means and standard deviations, skewness, and kurtosis of the distributions of each dimension, namely self-efficacy (efficacy in student engagement, efficacy in instructional strategies, and efficacy in classroom management), burnout (exhaustion and disengagement), irrational beliefs (self-downing, authoritarianism, demands for justice, and low frustration tolerance), and innovative work behavior (idea generation, idea promotion and idea realization, and idea sustainability), respectively.

Table 2 presents the correlation matrix of the 13 dimensions, which were the inputs for performing structural equation modeling (SEM). The results of the structural equation modeling (SEM) are shown in Table 3 and Table 4, where the factor loadings and the regression coefficients are presented, respectively.

The SEM fit indices were satisfactory, showing a statistically significant model that can describe and explain the associations among the variables predicting IWB. Here, [*χ^2^*(43) = 340,767, *p* < 0.001, *TLI* = 0.920, *CFI* = 0.956, *GFI* = 0.998, *NNFI* = 0.920, *RMSEA* = 0.085 (0.077–0.093), *SRMR* = 0.057].

**Figure 2:**
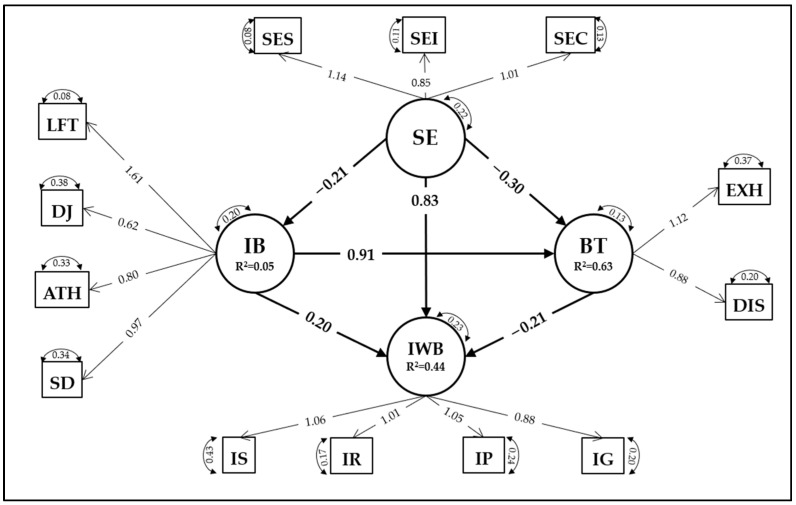
The structural equation model predicting innovative work behavior [*TLI* = 0.920, *CFI* = 0.956, *RMSEA* = 0.085, *SRMR* = 0.057].

The successful SEM model is depicted in Figure 2 and suggests that there is direct positive effect from SE on IWB (*b* = 0.833, *p* < 0.001) and two indirect effects, namely a negative effect via BT on IWB (*b* = −0.210, *p* < 0.001) and a positive effect via IB on IWB (*b* = 0.192, *p* < 0.001). In addition, SE affects BT negatively (*b* = −0.303, *p* < 0.001) and SE also negatively affects IB (*b* = −0.209, *p* < 0.001), while IB affects BT positively (*b* = 0.912, *p* < 0.001). Mediation analysis showed that the effects under investigation [SE → BT → IWB and SE → IB → IWB] are statistically significant (*p* < 0.001). To this end, the hypothesized SEM proved satisfactorily explanatory to the teachers’ IWB [IWB (*R^2^* = 0.441); BT (*R^2^* = 0.626); IB (*R^2^* = 0.045)].

## 4. Discussion

### 4.1. Discussion and Interpretation of the Findings

The purpose of this paper was to investigate IWB as a function of SE, BT, and IB. An attempt was also made to examine potential relationships among the aforementioned independent variables. Thus, the proposed SEM model revealed the mediating role of BT and IB, indicating additional indirect effects on IWB.

As expected, and in line with previous research [4,15], teachers’ SE is a main predictor of their IWB, with a positive effect (*H*_1_). The more confident teachers feel about their personal abilities, the greater their behavioral orientation will be towards innovation. Teachers with low competence in managing their classrooms, strengthening students’ engagement, or adapting their teaching strategies, are less likely to exhibit innovative behavior.

Furthermore, it was found that SE has significant negative effects on the teachers’ BT and IB (*H*_2_). Here, BT is negatively influenced by teachers’ SE; that is, higher SE could prevent emotional exhaustion and their gradual elimination and disengagement from work, a finding consistent with the theory and with previous and more recent studies [37,75,76]. Moreover, SE seems to reduce IB. This finding is interpretable, considering that IB are the kind of beliefs that could be characterized by a lack of rationality. A misconception about one’s personal abilities can be a factor fortifying IB, while a mutual causality could also be considered [30,33].

Consequently, BT has a negative effect on teachers’ IWB (*H*_5_); that is, the latter is significantly reduced when teachers feel emotionally exhausted and are relieved by their disengagement. The demanding schoolwork environment is undoubtedly an enhancing work-stress factor and is likely causing the BT of teachers [50]. As a result, any effort to foster innovation becomes difficult, as teachers do not feel satisfied with their work and there is a strong lack of willingness to offer anything new, different, and innovative [77]. The firm conclusion drawn from a plethora of research endeavors is that BT is a deterrent to the development of IWB [78,79].

The last variable whose role was examined via the SEM model is IB, which was found to significantly affect IWB and BT as well. Specifically, regarding BT, IB have a positive effect on the former (*H*_3_); that is, it is likely that more IB fortify exhaustion and disengagement, given that they comprise a factor which also enhances work stress, as has been affirmed in recent studies [77]. Their negative impact on the emotional intelligence and psychological resilience of educators reduces their job satisfaction, and evidently facilitates increasing BT.

An interesting finding, however, is that IB seem to have a positive effect on IWB, which does not support *H*_4_. From the correlation matrix (Table 2), it could be observed that the relationships among the dimension of IB and IWB are not consistent in terms of sign and strength. That is, they vary from positive to negative values, indicating a waving possible net effect, which is not predicted when considering the bivariate cases. The mathematical formulation of the SEM concludes on a positive net effect, which in this work is trusted as the emergent effect holding within the proposed model. Beyond the empirical evidence, some theoretical comments are further provided regarding the role of IB within the teacher self-regulation process.

Teachers’ additional fear of dealing successfully with new and unfamiliar situations, due to increased job demands, caused by curriculum innovations, encourages the development of IB. One would, therefore, expect the increased IB to deter teachers’ IWB. The finding seems to differ from the expected result based on the prevailing view of IB. Nevertheless, it is interpretable, as IB bring mainly dysfunctional consequences, without excluding the provocation of functional responses. After all, IB are an individual characteristic [6]; they have different consequences and manifest in different forms, affecting each person uniquely. As such, IB are not bipolar in nature, as they can manifest in a wide range of tensions and interact with each other through a network of complex relationships [80,81]. Their combined power is what determines whether the way in which they act will be functional or dysfunctional [82], while individuals under different circumstances might possess IB of varying strengths [83]. The peculiar behavior of IB, appearing as positive or negative predictors, is not necessarily a contradiction. An application within a complex and dynamic system perspective provides a cogent interpretation of the ambiguous behavior of dysfunctional myths, revealing their role as bifurcation factor that induces nonlinear effects and introduces uncertainty in the underlying processes [60].

Finally, the proposed SEM model indicates that SE has a direct effect on IWB, but also indirectly, as IB and BT function as mediators, supporting *H*_6_. In addition, IB directly affect IWB, but can also have an indirect effect, as BT acts as a mediating factor. In conclusion, significant relationships were identified between all the variables examined in the present study, giving the proposed model interpretive power. Teachers’ IWB is part of a field that has not yet been fully explored and seems to be influenced by a multiplicity of individual characteristics, some of which are examined for the first time in this endeavor, expanding our knowledge on the subject matter.

The findings of this research, besides the theoretical value of contributing to the literature regarding the school context, also have significant implications for practice. Since teachers, as with any person, possess beliefs which influence the way they think and act, knowing the individual differences that affect the behavior in question, the school management should intervene and mediate to achieve the changes that lead to the promotion of IWB. More specifically, enhancing teachers’ SE that is a key factor in professional or non-professional development, and is the first priority for strengthening teachers’ IWB. Furthermore, interventions programs, where issues related to irrational believes are analyzed, could be targeted for preventing or reducing IB to the extent that they add to burnout. It is worth noting, however, that, in this research, the dual role of IB was highlighted, which seem to increase BT, but also to strengthen the IWB. This peculiar behavior of IB needs special attention, given that their presence does not necessarily imply the development of dysfunctional outcomes and consequences but can also bring about functional responses. Finally, BT appears to be a suppressive factor regarding IWB. Alternative ways of teaching in connection with professional training would reduce the BT.

### 4.2. Limitations

This paper has some limitations that need to be considered. In the collection of data, self-report questionnaires were used, while a convenience sampling procedure was followed. The proposed model, being tested for the first time, needs to be further confirmed with additional data sets and probably improved with additional variables which have been ignored in the current design. Finally, limitations exist because of the use of linear modelling and its restrictions imposed by the distributional presupposition, which are not completely satisfied, while the linear approach might have not revealed all the possible relationships between the variables in question.

### 4.3. Future Research

The present study focused on highlighting the possible factors that influence teachers’ IWB. However, the continuous development of innovation in the school environment, clearly designates the need for further investigations. Factors, such as motivation, emotions, student performance, and job satisfaction, may need further consideration.

An interesting extension of the present study is the examination of these variables with different participants. Teachers in other levels of education, such as high schools and preschools, are likely to demonstrate different findings regarding the factors that influence their IWB. The different work environments, as well as the different students’ ages with corresponding demands, might reasonably lead to differentiated and surprising results.

Finally, the hypotheses tested in the present work can be re-examined by applying nonlinear analyses (e.g., [60]). This additional investigation of the relationships between the variables under study will open a new perspective in the field that can provide a better understanding of the interactive processes and their dynamics governing the school environment.

## 5. Conclusions

The multivariate analysis via structural equation model demonstrated the roles and the effects of independent factors on teachers’ IWB. The direct effect of self-efficacy in combination to the effects of irrational beliefs and burnout, acting as mediators, illustrate the complexity of innovative work behavior, and the multiplicity of factors determining teachers’ performance. The model, of course, does not imply that the outcome from their interactions is a linear sum of all contributing components, but it provides an insight about the combined effect, suggesting which factors have to be reinforced and which have to be reduced to attain the desirable behavior. The empirical evidence supports the theoretical conjectures, and school management is informed about the effective conditions that have to be offered to employees. After all, teachers are a professional group, which clearly requires the adoption and development of innovations for the success of its smooth commission.

## Figures and Tables

**Table 1:** Descriptive statistics.

Scale	Mean	Std. Deviation	Cronbach’s a	Skewness	Kurtosis
SEC	3.854	0.602	0.782	−0.301	0.539
SEI	4.260	0.516	0.757	−0.656	1.430
SES	4.020	0.606	0.753	−0.431	0.611
DIS	1.968	0.693	0.751	0.893	0.857
EXH	2.695	0.903	0.815	0.241	−0.443
SD	2.710	0.733	0.745	0.081	−0.127
ATH	2.415	0.682	0.760	0.468	0.151
DJ	3.956	0.675	0.684	−0.692	0.676
LFT	2.323	0.799	0.735	0.432	0.038
IG	3.801	0.725	0.825	−0.622	0.667
IP	3.482	0.838	0.891	−0.474	0.268
IR	3.781	0.771	0.911	−0.684	0.898
IS	3.059	0.948	0.854	−0.096	−0.579

Abbreviations are as follows: SEC, self-efficacy for classroom management; SEI, self-efficacy for instructional strategies; SES, self-efficacy for student engagement; DIS, disengagement; EXH, exhaustion; SD, self-downing; ATH, authoritarianism; DJ, demands for justice; LFT, low frustration tolerance; IG, idea generation; IR, idea realization; IP, idea promotion; IS, idea sustainability.

**Table 2:** Correlation matrix of the 13 dimensions.

	SEC	SEI	SES	DIS	EXH	SD	ATH	DJ	LFT	IG	IP	IR	IS
**SEC**	1												
**SEI**	0.604 **	1											
**SES**	0.662 **	0.673 **	1										
**DIS**	−0.298 **	−0.246 **	−0.343 **	1									
**EXH**	−0.170 **	−0.178 **	−0.166 **	0.572 **	1								
**SD**	−0.144 **	−0.104 **	−0.154 **	0.326 **	0.369 **	1							
**ATH**	−0.136 **	−0.108 **	−0.238 **	0.345 **	0.271 **	0.532 **	1						
**DJ**	0.134 **	0.109 **	0.069 *	0.184 **	0.293 **	0.311 **	0.226 **	1					
**LFT**	−0.126 **	−0.187 **	−0.169 **	0.504 **	0.557 **	0.483 **	0.419 **	0.362 **	1				
**IG**	0.389 **	0.402 **	0.461 **	−0.279 **	−0.117 **	−0.097 **	−0.176 **	0.173 **	−0.124 **	1			
**IP**	0.426 **	0.388 **	0.466 **	−0.243 **	−0.149 **	−0.072 *	−0.087 **	0.129 **	−0.083 **	0.815 **	1		
**IR**	0.431 **	0.453 **	0.487 **	−0.274 **	−0.116 **	−0.088 **	−0.139 **	0.125 **	−0.122 **	0.796 **	0.809 **	1	
**IS**	0.397 **	0.313 **	0.410 **	−0.249 **	−0.129 **	−0.063	−0.116 **	0.040	−0.056	0.681 **	0.776 **	0.683 **	1

* *p* < 0.05, ** *p* < 0.01. Abbreviations are as follows: SEC, self-efficacy for classroom management; SEI, self-efficacy for instructional strategies; SES, self-efficacy for student engagement; DIS, disengagement; EXH, exhaustion; SD, self-downing; ATH, authoritarianism; DJ, demands for justice; LFT, low frustration tolerance; IG, idea generation; IR, idea realization; IP, idea promotion; IS, idea sustainability.

**Table 3:** Factor loadings.

						95% Confidence Interval		Standardized		
Latent	Indicator	Estimate	Std. Error	z-Value	*p*	Lower	Upper	All	LV	Endo
BT	DIS	0.882	0.026	34.484	<0.001	0.832	0.932	0.761	0.527	0.761
	EXH	1.118	0.026	43.697	<0.001	1.068	1.168	0.740	0.668	0.740
IB	SD	0.968	0.049	19.728	<0.001	0.872	1.064	0.611	0.447	0.611
	ATH	0.801	0.051	15.629	<0.001	0.700	0.901	0.542	0.370	0.542
	DJ	0.620	0.058	10.720	<0.001	0.507	0.734	0.425	0.287	0.425
	LFT	1.611	0.059	27.335	<0.001	1.495	1.726	0.932	0.744	0.932
IWB	IG	0.883	0.026	34.587	<0.001	0.833	0.933	0.785	0.569	0.785
	IP	1.050	0.023	45.552	<0.001	1.005	1.095	0.808	0.677	0.808
	IR	1.011	0.027	37.132	<0.001	0.958	1.065	0.846	0.652	0.846
	IS	1.056	0.036	29.321	<0.001	0.985	1.126	0.718	0.681	0.718
SE	SEC	1.014	0.033	30.481	<0.001	0.948	1.079	0.792	0.476	0.792
	SEI	0.846	0.030	27.909	<0.001	0.786	0.905	0.771	0.397	0.771
	SES	1.141	0.030	37.430	<0.001	1.081	1.200	0.884	0.536	0.884

Abbreviations are as follows: BT, burnout; IB, irrational beliefs; IWB, innovative work behavior; SE, self-efficacy; SEC, self-efficacy for classroom management; SEI, self-efficacy for instructional strategies, SES, self-efficacy for student engagement; DIS, disengagement; EXH, exhaustion; SD, self-downing; ATH, authoritarianism; DJ, demands for justice; LFT, low frustration tolerance; IG, idea generation; IR, idea realization; IP, idea promotion; IS, idea sustainability.

**Table 4:** Regression coefficients.

						95% Confidence Interval		Standardized		
Predictor	Outcome	Estimate	Std. Error	z-Value	*p*	Lower	Upper	All	LV	Endo
SE	BT	−0.303	0.033	−9.126	<0.001	−0.368	−0.238	−0.239	−0.239	−0.239
IB	BT	0.912	0.042	21.914	<0.001	0.830	0.993	0.705	0.705	0.705
SE	IB	−0.209	0.035	−6.045	<0.001	−0.276	−0.141	−0.212	−0.212	−0.212
BT	IWB	−0.210	0.038	−5.486	<0.001	−0.285	−0.135	−0.195	−0.195	−0.195
IB	IWB	0.192	0.052	3.724	<0.001	0.091	0.294	0.138	0.138	0.138
SE	IWB	0.833	0.025	32.828	<0.001	0.783	0.883	0.607	0.607	0.607

Abbreviations are as follows: BT, burnout; IB, irrational beliefs; IWB, innovative work behavior; SE, self-efficacy.

## Data Availability

The data presented in this study are available on request from the corresponding author.
